# Identification of the Autochaperone Domain in the Type Va Secretion System (T5aSS): Prevalent Feature of Autotransporters with a β-Helical Passenger

**DOI:** 10.3389/fmicb.2017.02607

**Published:** 2018-01-05

**Authors:** Maricarmen Rojas-Lopez, Mohamed A. Zorgani, Lawrence A. Kelley, Xavier Bailly, Andrey V. Kajava, Ian R. Henderson, Fabio Polticelli, Mariagrazia Pizza, Roberto Rosini, Mickaël Desvaux

**Affiliations:** ^1^Université Clermont Auvergne, INRA, UMR454 MEDiS, Clermont-Ferrand, France; ^2^GSK, Siena, Italy; ^3^Structural Bioinformatics Group, Imperial College London, London, United Kingdom; ^4^Institut National de la Recherche Agronomique, UR346 Epidémiologie Animale, Saint Genès Champanelle, France; ^5^CRBM UMR5237 CNRS, Institut de Biologie Computationnelle, Université Montpellier, Montpellier, France; ^6^Institute of Microbiology and Infection, University of Birmingham, Birmingham, United Kingdom; ^7^Department of Sciences, National Institute of Nuclear Physics, Roma Tre University, Rome, Italy

**Keywords:** protein secretion system, Autotransporters, Type V secretion system, Outer membrane proteins, Protein translocation, Autochaperone domain, diderm-LPS Gram-negative bacteria

## Abstract

Autotransporters (ATs) belong to a family of modular proteins secreted by the Type V, subtype a, secretion system (T5aSS) and considered as an important source of virulence factors in lipopolysaccharidic diderm bacteria (archetypical Gram-negative bacteria). While exported by the Sec pathway, the ATs are further secreted across the outer membrane via their own C-terminal translocator forming a β-barrel, through which the rest of the protein, namely the passenger, can pass. In several ATs, an autochaperone domain (AC) present at the C-terminal region of the passenger and upstream of the translocator was demonstrated as strictly required for proper secretion and folding. However, considering it was functionally characterised and identified only in a handful of ATs, wariness recently fells on the commonality and conservation of this structural element in the T5aSS. To circumvent the issue of sequence divergence and taking advantage of the resolved three-dimensional structure of some ACs, identification of this domain was performed following structural alignment among all AT passengers experimentally resolved by crystallography before searching in a dataset of 1523 ATs. While demonstrating that the AC is indeed a conserved structure found in numerous ATs, phylogenetic analysis further revealed a distribution into deeply rooted branches, from which emerge 20 main clusters. Sequence analysis revealed that an AC could be identified in the large majority of SAATs (self-associating ATs) but not in any LEATs (lipase/esterase ATs) nor in some PATs (protease autotransporters) and PHATs (phosphatase/hydrolase ATs). Structural analysis indicated that an AC was present in passengers exhibiting single-stranded right-handed parallel β-helix, whatever the type of β-solenoid, but not with α-helical globular fold. From this investigation, the AC of type 1 appears as a prevalent and conserved structural element exclusively associated to β-helical AT passenger and should promote further studies about the protein secretion and folding via the T5aSS, especially toward α-helical AT passengers.

## Introduction

Bacteria can secrete proteins by numerous molecular machineries. In this field, it is of key importance to differentiate export (protein transport across the cytoplasmic membrane) from secretion (protein transport from inside to outside the cell) systems (Desvaux et al., 2004b; Economou et al., 2006), especially in lipopolysaccharidic diderm (LPS-diderm) bacteria (archetypical Gram-negative bacteria) (Desvaux et al., 2009; Sutcliffe, 2010; Chagnot et al., 2013). While Sec (Secretion) and Tat (Twin-arginine translocation) systems constitute the two major export pathways, nine protein secretion systems numbered from Type I to Type IX (T1SS-T9SS) are currently recognised in the LPS-diderm bacteria, which enable protein transport across the outer most biological membrane. Among them, the T5SS most certainly secrete the most diverse range of effectors, thus constituting a premium source of virulence factors (Henderson and Desvaux, 2004; Henderson et al., 2004). The T5SS is currently subdivided into the (i) autotransporters (T5aSS), (ii) two-partner passenger-translocators (T5bSS), (iii) trimeric autotransporters (T5cSS), (iv) hybrid autotransporters (T5dSS), and (v) inverted autotransporters (T5eSS) (Desvaux et al., 2003; Leo et al., 2012).

The T5SS can be broadly defined as protein transport across the asymmetric LPS-containing outer membrane (OM) *via* a β-barrel to complete the secretion, which is first initiated by protein export *via* the Sec machinery for cytoplasmic inner membrane (IM) transit (Henderson et al., 2004; Leo et al., 2012). Nonetheless, several periplasmic chaperones, the BAM (β-barrel assembly machinery) and TAM (translocation and assembly module) complexes take part to the OM secretion process (Leyton et al., 2012; Selkrig et al., 2012). Regarding the T5aSS, the autotransporters (ATs) have a modular architecture constituted of three major regions, (i) a N-terminal signal peptide (SP), (ii) a central passenger, and (iii) a C-terminal translocator (Desvaux et al., 2004a; Drobnak et al., 2015). The SP targets the proteins to the IM before being cleaved off after export *via* Sec. Some autotransporter SPs exhibit a highly conserved domain called ESPR (Extended Signal Peptide Region) (Desvaux et al., 2006), which influence IM and OM translocation but whose exact function remains unclear (Desvaux et al., 2007; Jong and Luirink, 2008). The passenger is secreted across the OM and corresponds to the effector, which is either displayed at the bacterial cell surface or further released into the extracellular milieu. The passengers are generally believed to exhibit a β-helical structure (Jenkins et al., 1998; Kajava and Steven, 2006) but this is not systematic, e.g. EstA (Esterase Autotransporter) has a globular fold dominated by α-helices and loops, which is regarded as a general feature in the lipase/esterase ATs (LEATs) (Van Den Berg, 2010; Celik et al., 2012). In the β-helical passenger of ATs, different types of a β-solenoid motif are currently recognised as either displaying a triangular or L-shape coil cross-sections (Kajava and Steven, 2006). The translocator forms the translocation unit (TU) composed of an α-helical linker and a β-barrel domain, through which the passenger is transported across the OM (Oomen et al., 2004).

The investigation of secretion and folding of BrkA (*Bordetella r*esistance to killing) evidenced the importance of an autochaperone (AC) domain localised at the C-terminal region of the passenger was evidenced (Oliver et al., 2003). Besides BrkA, requirement of the AC for proper passenger secretion and cell-surface folding was supported by several investigations in AIDA-I (*Escherichia coli A*dhesin Involved in Diffuse Adherence I) (Berthiaume et al., 2007), EspP (Extracellular serine protease precursor) (Velarde and Nataro, 2004), Hbp (Hemoglobin-binding protease) (Soprova et al., 2010), IcsA (Intra-cellular spread protein A) (May and Morona, 2008), Pet (Plamid-encoded toxin) (Dutta et al., 2003) and Ssp (*Serratia marcescens* serine protease) (Ohnishi et al., 1994). When mutated, secretion of BrkA could be rescued with the AC supplied in *trans* (Oliver et al., 2003), which was also demonstrated in Ssp (Ohnishi et al., 1994), EspP and Pet (Dutta et al., 2003). This suggested that the AC provides a template-induced folding mechanism for the passenger. More recently, the crystal structure of the AC of IcsA was resolved and this clearly appeared to exhibit a characteristic β-sandwich fold (Kuhnel and Diezmann, 2011). However, considering it was functionally characterised and identified only in a handful of autotransporters, suspicion recently fell on the commonality and conserved nature of the AC as a sequence element (Drobnak et al., 2015). Still, the identification of this domain could be limited by classical BLAST search due to sequence divergence (Altschul and Koonin, 1998).

This prompted us to investigate the prevalence of the AC in the T5aSS. Taking advantage of the resolved three-dimensional structure of the AC from IcsA, this domain was searched by structural alignment, first, among all passengers experimentally resolved by crystallography, before searching against a database of well-defined and genuine ATs. From there, phylogenetic analysis revealed the organisation of the AC family, whereas proteogenomic analysis pinpointed that the AC was systemically associated with passengers exhibiting a β-helix fold but not a globular fold like EstA. Taken together, the AC appears as a conserved domain exclusively present in ATs with a β-helical passenger.

## Materials and methods

### Structural alignment

Superimposition of one structure against another was performed using PyMOL v1.7.4. The atomic coordinates and structure information of the proteins of interest were recovered from the Protein Data Bank (PDB) (Berman et al., 2000a,b). The structures were aligned to minimise the RMSD (Root Mean Square Deviation) between the aligned atoms (C-alfas). For multiple sequence alignment, a special mode of T-Coffee (Notredame et al., 2000; Di Tommaso et al., 2011; Magis et al., 2014) was used to incorporate structural information, i.e. Expresso (Armougom et al., 2006), which is an extension of 3D-Coffee where structure based alignment is used as a template for realigning the original sequences, which results in a structure-based multiple sequence alignment (O'sullivan et al., 2004). The alignment was then visualised with ENDscript, which combines both primary sequence and secondary structure alignment (Gouet et al., 2003; Robert and Gouet, 2014).

### Search for domain homologs

The AC domains were also identified in autotransporters by submitting passenger sequences to Phyre v2.0 for automated modeling. The dataset included the 1523 well-defined and genuine autotransporters identified by the twin-HMM autotransporter procedure designed by Celik et al. (2012). In parallel, using the refined AC structures here defined from structural alignments of IcsA, EspP, Hbp, Pet, pertactin P69, Hap, and IgA1 (PDB files provided as Supplementary Materials), the presence of the AC in other autotransporters was searched using Phyre in reverse, i.e., BackPhyre (Kelley and Sternberg, 2009; Kelley et al., 2015). To achieve a high degree of reliability with respect to the predicted domain fold and modeling of the protein core at high accuracy (<4 Å RMSD from native, true structure), only structural matches with a high level of confidence (>90%) were considered.

### Phylogenetic analysis of protein sequences

The ACs identified by structural alignment with Phyre/BackPhyre were aligned with T-Coffee in the expresso mode, using PDB files restricted to the AC domains of IcsA (PDB: 3ML3; D_606_–L_720_), EspP (PDB: 3SZE; D_869_–I_979_), Hbp (PDB: 1WXR; N_948_–L_1056_), Pet (PDB: 4OM9; N_865_–I_974_), P69 (PDB: 1DAB; D_444_–L_556_), Hap (PDB: 3SYJ; D_830_–L_964_), and IgA1 (PDB: 3H09; D_865_–L_977_) to seed the alignment. A BioNJ tree (Gascuel, 1997) based on observed divergences between pairs of sequences was obtained using SplitsTree (Kloepper and Huson, 2008). The most relevant clusters, i.e., monophyletic groups or clades, were identified and selected based on splits showing bootstrap values above 80% over 1,000 pseudo-replicates.

### Identification of functional motifs and secondary structures

Functional motifs were searched using InterProScan v5.22 (Jones et al., 2014) and interrogating InterPro (IPR) v61.0 database (Finn et al., 2017), which includes CATH-Gene3D v4.1 (Sillitoe et al., 2015; Lam et al., 2016), CDD (Marchler-Bauer et al., 2017), MobiDB v2.3.2014.07 (Potenza et al., 2015), HAMAP (Pedruzzi et al., 2015), PANTHER v11.1 (Mi et al., 2017), Pfam v30.0 (Finn et al., 2016), PIRSF (Wu et al., 2004), PRINTS (Attwood et al., 2003), ProDom v2012.1 (Servant et al., 2002), PROSITE v2017.01 (Sigrist et al., 2013), SFLD (Akiva et al., 2014), SMART v7.0 (Schultz et al., 1998), SUPERFAMILY v1.75 (Wilson et al., 2009), TIGRFAMs v15.0 (Haft et al., 2003). Besides structure modeling using Phyre v2, β-helix folds were predicted with BetaWrap, using rung profile (3–7 rungs) (Bradley et al., 2001), as well as PfScan to identify the types of β-solenoid motif (Kajava and Steven, 2006).

## Results

### Identification of the autochaperone domain in autotransporter passengers with a resolved three-dimensional structure

**Figure 1:**
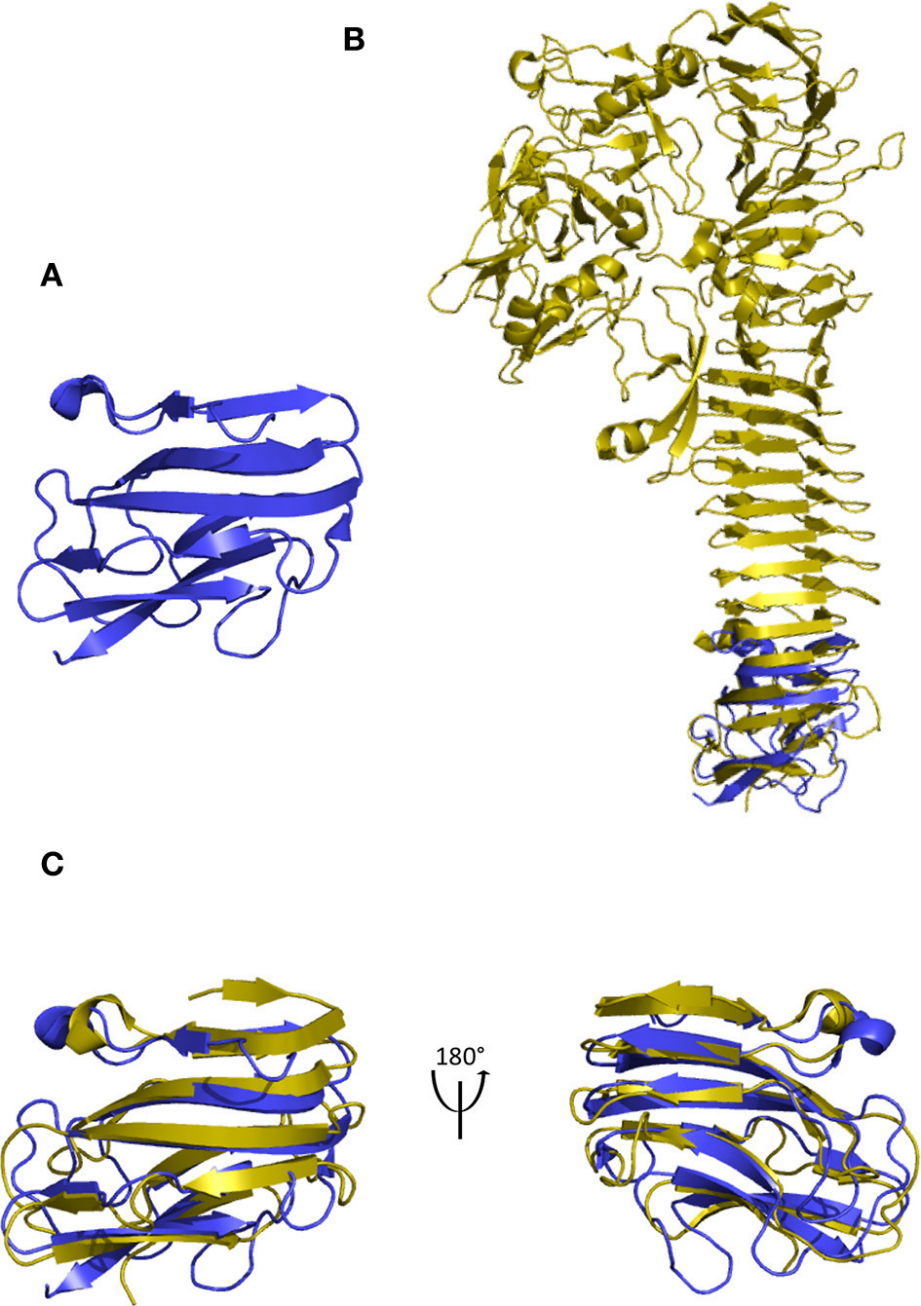
Structural identification of the autochaperone (AC) in resolved structures of autotransporter passengers with EspP as a case study. **(A)** Refined AC of IcsA (PDB: 3ML3; L_606_–D_720_). **(B)** Structural identification of the AC domain (blue) in the C-terminal region of the EspP passenger (PDB: 3SZE; yellow). **(C)** Superimposed ACs of IcsA (D_606_–L_720_) and of EspP (D_869_–A_979_).

**Figure 2:**
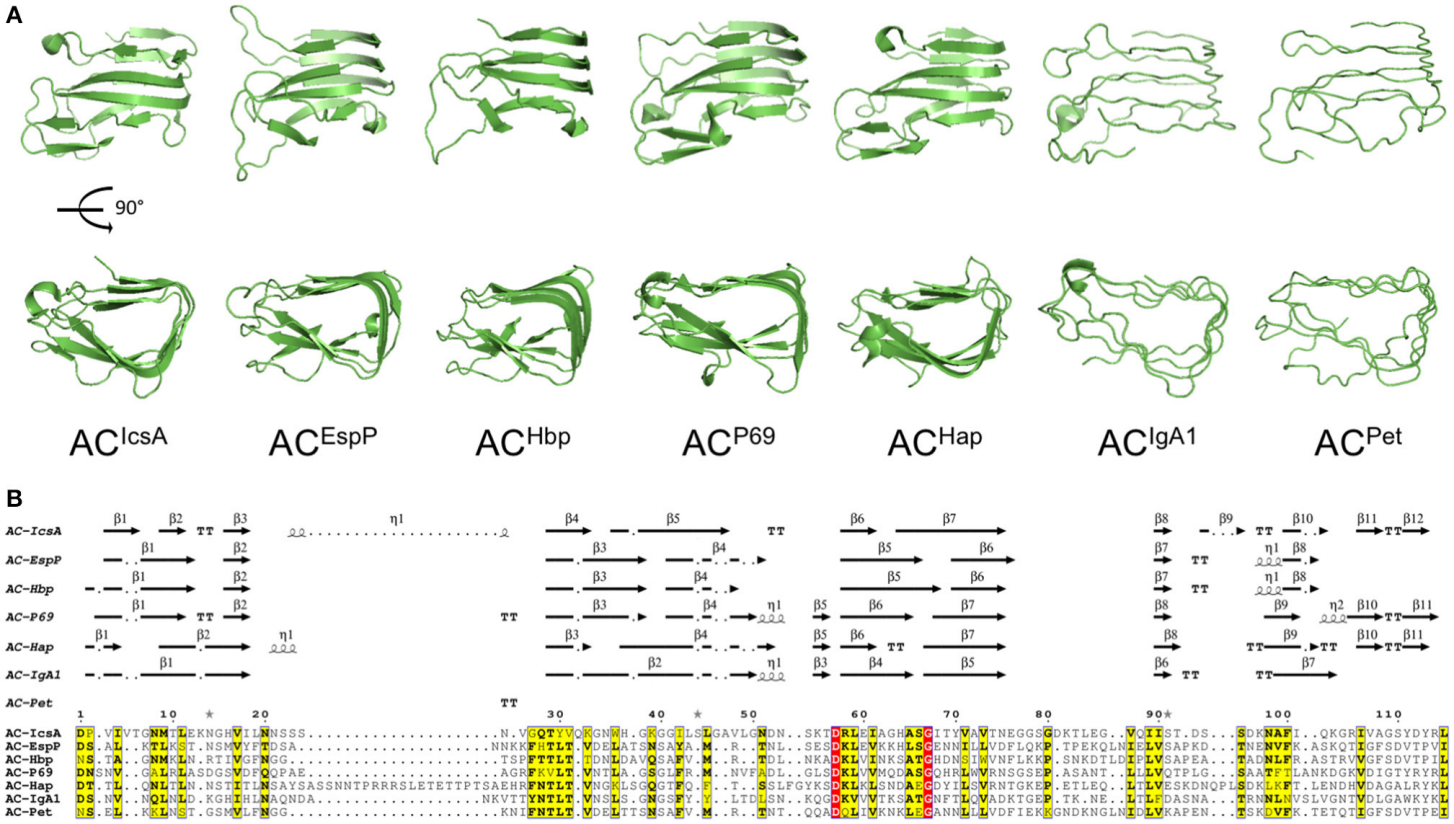
The autochaperone (AC) domains identified in autotransporter passengers with a resolved structure. **(A)** Three-dimensional structures of the resolved AC domains. **(B)** Multiple primary sequence alignment of the resolved AC domains. β-strands are indicated with arrows, α-helices are indicated with curls, and turns with T. AC^IcsA^ (PDB: 3ML3; D_606_–L_720_), AC^EspP^ (PDB: 3SZE; D_869_–I_979_), AC^Hbp^ (PDB: 1WXR; N_948_–L_1056_), AC^P69^ (PDB: 1DAB; D_444_–L_556_), AC^Hap^ (PDB: 3SYJ; D_830_–L_964_), AC^IgA1^ (PDB: 3H09; D_865_–L_977_), and AC^Pet^ (PDB: 4OM9; N_865_–I_974_). The coordinates of all AC structures here analysed are provided as Supplementary Materials as PDB files.

While the autochaperone (AC) was experimentally identified in BrkA, AIDA-I, EspP, Hbp, Pet, and Ssp, neither the structure of the passenger of BrkA, AIDA-I nor Ssp has been experimentally resolved by crystallography, or by any other mean, contrary to EspP (PDB: 3SZE) (Khan et al., 2011), Hbp (PDB: 1WXR) (Otto et al., 2005), and Pet (PDB: 40M9) (Domingo Meza-Aguilar et al., 2014) passengers. Using the structure of the AC of IcsA as reference (AC^IcsA^; PDB: 3ML3) (Kuhnel and Diezmann, 2011), identification of the AC in the C-terminal part of EspP, Hbp, and Pet was first attempted. While the resolution of the secondary structure of Pet was low in the C-terminal region of the passenger (PDB: 40M9) and the RMSD was slightly high for Hbp when superimposed to AC^IcsA^ (RMSD > 4 Å over 54 atoms), the 3D-structure of the C-terminal part of EspP (D_869_–I_979_) superimposed onto residues D_606_–L_720_ of IcsA (Figure 1) with RMSD of 2.51 Å over 43 atoms (Table 1). The structural region here identified as the AC^EspP^ completely agrees with previous experimental investigations by functional mutagenesis where the AC was identified within the 821–997 region of EspP (Dutta et al., 2003; Velarde and Nataro, 2004; Skillman et al., 2005; Brockmeyer et al., 2009). Similarly, AC^Pet^ (N_865_–I_974_) was here identified within the 819–992 region of Pet, functionally characterised as an autochaperone (Dutta et al., 2003) and within the 950–1,048 region for the AC^Hbp^ (N_948_–L_1056_) (Soprova et al., 2010). Using the AC^IcsA^ structure (D_606_–L_720_), this domain was structurally aligned within the C-terminal region of all other crystallised autotransporter passengers, namely pertactin P69 (PDB: 1DAB), Hap (PDB: 3SYJ), IgA1 (PDB: 3H09), EstA (PDB: 3KVN), Ag43 (PDB: 4KH3), and VacA (PDB: 2QV3). Except for EstA, Ag43, and VacA, an AC could be identified in all other resolved AT passengers. Following structure alignment, the RMSD varied between 1.51 Å for AC^IcsA^ vs. AC^P69^ and 3.40 Å for AC^IcsA^ vs. AC^IgA1^ (Table 1). Once identified in each passenger, these AC domains were further structurally aligned one with another and appeared to superimpose with RMSD ranging from 0.44 to 2.97 Å for AC^Hbp^ vs. AC^EspP^ and for AC^P69^ vs. AC^Pet^ respectively (Table 1). Of note, the AC^Hbp^ superimposed to the AC of these other ATs with RMSD systematically lower than 2 Å (Table 1). With a size ranging from 109 to 135 amino acid (a.a.) residues, the AC domains displayed a conserved structure forming coils of parallel and anti-parallel β-sheets and a couple of short α-helices (Figure 2A). For IgA1 and Pet, however, the resolution of the structure in this region was not high enough to provide information on the secondary structures. Sequence similarities of the identified AC were further confirmed following multiple sequence alignment incorporating structural information, where regions with β-strands or α-helices aligned one another in the different ACs (Figure 2B).

**Table 1:** RMSD values of superimposed AC domains present in the experimentally resolved autotransporters three-dimensional structures.

**RMSD^a^**	**AC^EspP^**	**AC^Hbp^**	**AC^P69^**	**AC^Hap^**	**AC^IgA1^**	**AC^Pet^**
AC^IcsA^	2.51 (43)	6.10 (67)	2.69 (59)	1.88 (81)	3.39 (74)	3.97 (97)
AC^Pet^	1.14 (83)	1.42 (70)	2.97 (33)	1.71 (54)	2.05 (54)	–
AC^IgA1^	2.48 (72)	1.11 (48)	2.02 (74)	0.54 (66)	–	–
AC^Hap^	2.18 (62)	1.27 (55)	0.89 (69)	–	–	–
AC^P69^	2.84 (69)	1.31 (54)	–	–	–	–
AC^Hbp^	0.44 (94)	–	–	–	–	–

a *RMSD (Root Mean Square Deviation) values (Å) with the number of superimposed atoms (in brackets) for each AC domains superimposed one with another and identified from the known three-dimensional structures of autotransporters IcsA (PDB: 3ML3; AC: D_606_–L_720_), EspP (PDB: 3SZE; AC: D_869_–I_979_), Hbp (PDB: 1WXR; AC: N_948_–L_1056_), Pet (PDB: 4OM9; AC: N_865_-I_974_), pertactin P69 (PDB: 1DAB; AC: D_444_–L_556_), Hap (PDB: 3SYJ; AC: D_830_–L_964_), and IgA1 (PDB: 3H09; AC: D_865_–L_977_)*.

### Distribution of the autochaperone among the T5aSS

**Figure 3:**
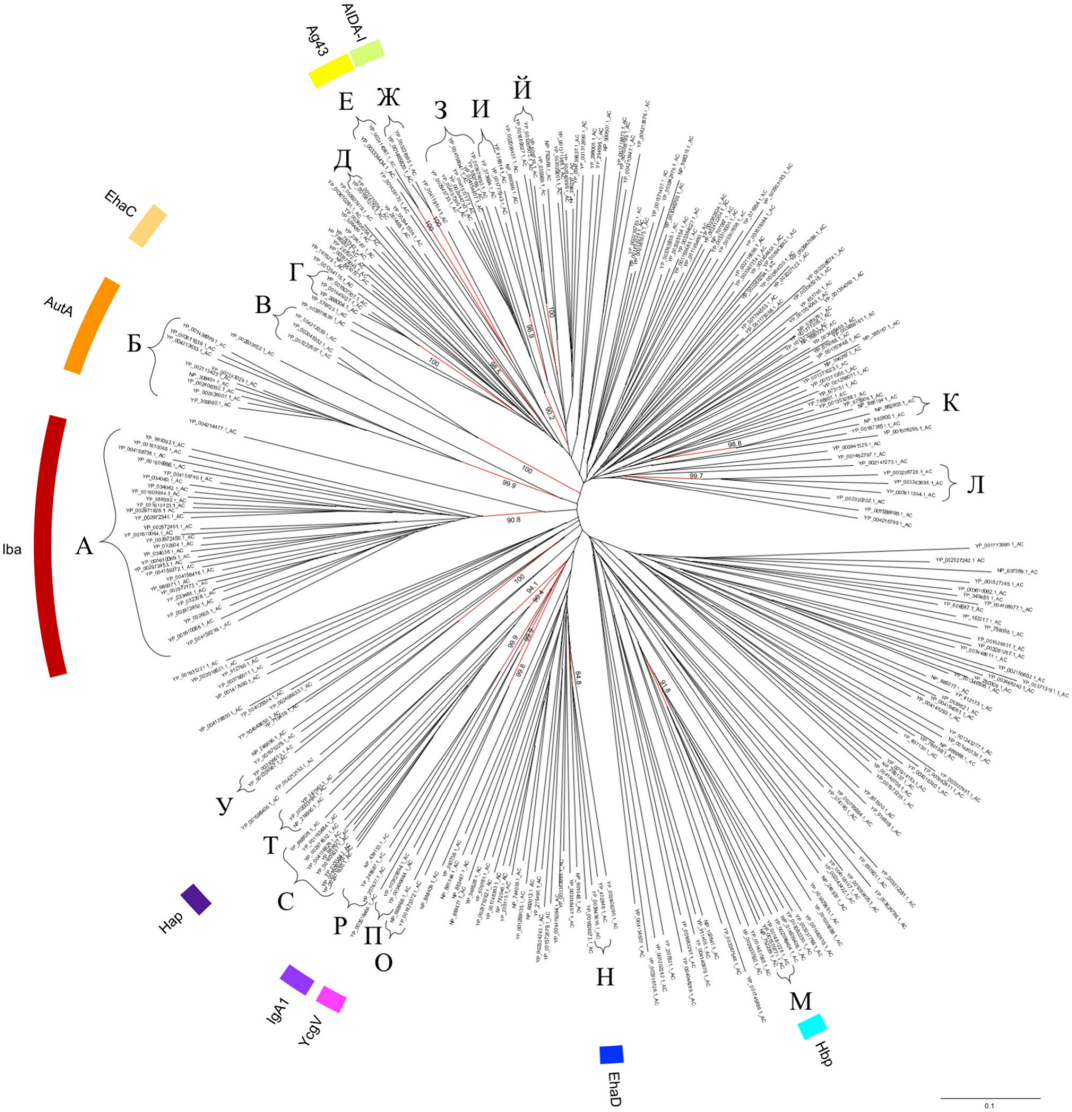
Phylogenetic tree of the AC domains in the T5aSS. Sequences of the AC domain were identified among the non-redundant well-defined dataset of 1523 autotransporters (Celik et al., 2012) following structural search using Phyre/BackPhyre. For legibility, the redundant and very closely related AC sequences were removed from the tree but are readily available in Table 1S. The scale bar represents the evolutionary distance, i.e., the number of substitutions per site.

To determine whether similar AC structures are present in other autotransporters, the well-defined ACs here identified from solved tertiary structures of passengers, namely AC^IcsA^ (D_606_–L_720_), AC^EspP^ (D_869_–I_979_), AC^Hbp^ (N_948_–L_1056_), AC^P69^ (D_444_–L_556_), and AC^Hap^ (D_830_–L_964_), were used as queries to search for similar structures using BackPhyre. In parallel, the AC domains were also identified by submitting the sequences corresponding to the passenger domain to Phyre for automated modeling. For data mining, the dataset was constituted of the well-defined and genuine ATs identified by Celik et al. (2012). Out of these 1523 sequences of autotransporters, an AC could be identified in 708 of them with confidence levels higher than 90.0%, which corresponded to 429 non-redundant and distinct sequences (Supplementary Material Table 1S). These AC domains have an average size of about 110 a.a. residues (mediane = 112 and mode = 115 a.a.) and were essentially present toward the C-terminal region of the passengers, adjacent to the translocator region at an average distance of about 100 a.a. residues upstream (mediane = 84 and mode = 67 a.a.). With respect to the ATs for which an AC was experimental identified but no structural information was available, an AC could indeed be identified in the C-terminal region of the BrkA and AIDA-I passengers but not Ssp. Regarding ATs for which the passenger structure was resolved but an AC domain could not be identified in the first instance, an AC was finally identified in the C-terminal part of the Ag43 passenger (position 525-638), i.e., a region that has not been crystallised (Heras et al., 2014), but this was not the case either for EstA or for VacA. Following multiple sequence alignment incorporating structural information, phylogenetic analysis revealed that the AC domains distribute into deeply rooted branches, from which 20 main clusters emerged (named according to cyrillic alphabet with phonetics in brackets), namely A [a], Б [b], B [v], Г [g], Д [d], E [je], Ж [ʒ], З [z], И [i], Й [j], К [k], Л [l], M [m], H [n], O [o], П [p], P [r], C [s], T [t], and У [u] (Figure 3), where clusters A and Б form the largest groups hosting some autotransporter members of Iba (Inducible *Bartonella* autotransporter) (Eicher and Dehio, 2012) and AutA (Auto-transporter A) groups respectively (Ait-Tahar et al., 2000). Clusters Г, Д, З, И, Й, K, Л, O, C, and У harbor ACs from autotransporters that have not been characterised yet. The resolved AC domains from IcsA, EspP, Pet, or pertactin P69 were not found in clusters but in deeply rooted branches, whereas the ACs from Hbp, IgA1, and Hap were part of clusters M, P, and T respectively (Figure 3). Considering other characterised autotransporters, clusters B, E, Ж, H, and П harbor ACs from EhaC, Ag43, AIDA-I, EhaD, and YcgV autotransporter members (Vo et al., 2017), whereas the ACs from BrkA and Pet were found in deeply rooted branches.

### The AC is exclusively associated with autotransporters exhibiting β-helical passengers

**Figure 4:**
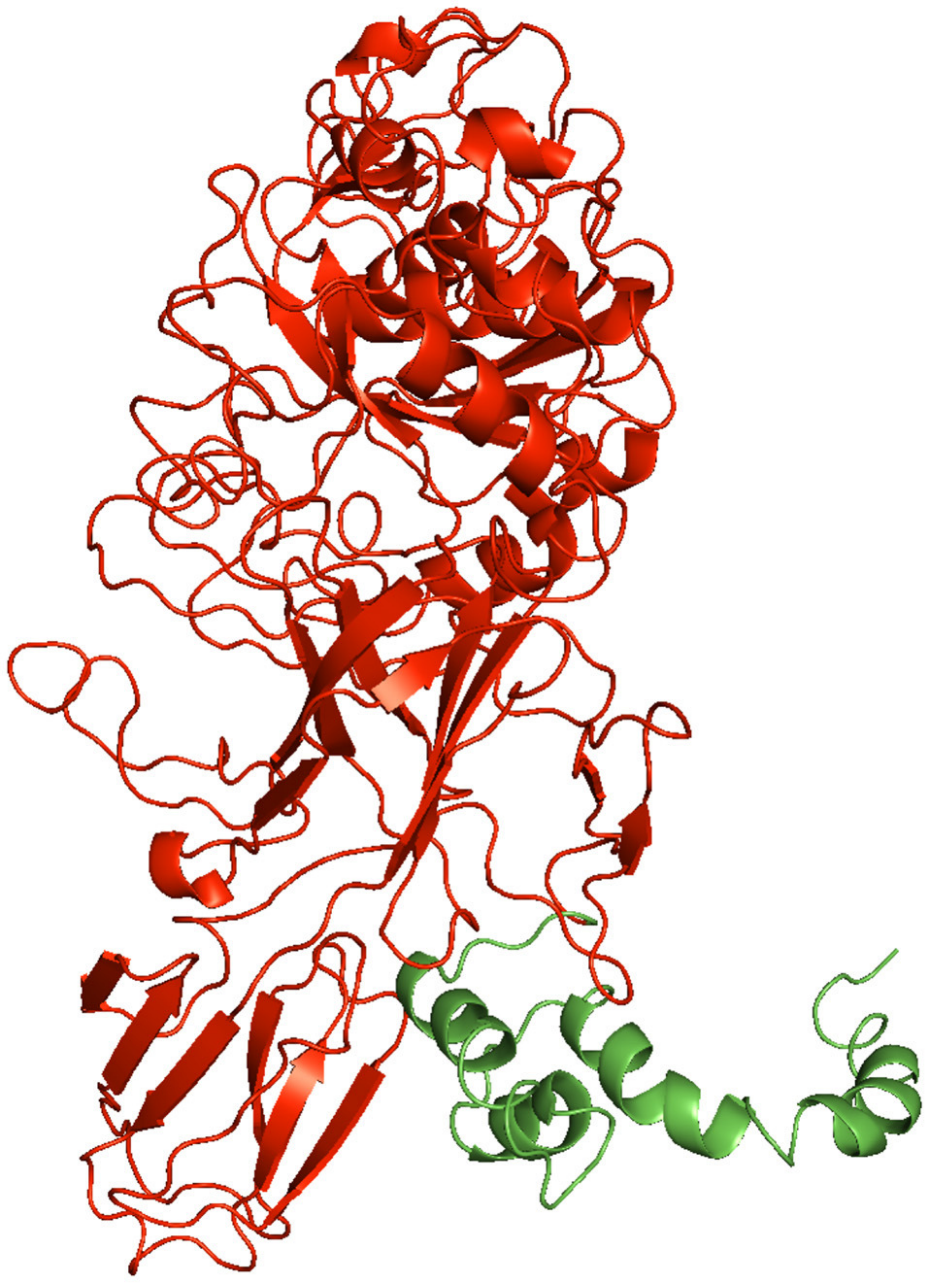
Three-dimensional structure modeling of the passenger of Ssp. The passenger sequence of Ssp (A_28_–G_716_) was submitted as query to Phyre using intensive mode. The structure model covers the 37–601 region (72% coverage of the query sequence) with a confidence level of 100.0% with the single highest scoring template (Subtilase family, PDB: 1R6V). The green and red colors depict the regions corresponding to the AC domain and functional domain of the passenger respectively. The structure of the AC domain was essentially modeled *ab initio* and must be regarded as unreliable. The coordinates of the Ssp passenger model are provided as Supplementary Material as a PDB file.

Looking for a correlation with the presence of the AC, functional genomic analysis of the ATs was performed (Supplementary Material Table 1S). First, an ESPR (IPR024973) could be predicted in the SP region of 134 ATs; in 84% of these an AC was also identified. Based on the functional motifs identified in the passengers, the ATs could be further classified into 6 main and distinct functional categories, *i.e*. the (i) protease autotransporters (PATs), (ii) self-associating autotransporters (SAATs), (iii) phosphatase/hydrolase autotransporters (PHATs), (iv) lipase/esterase autotransporters (LEATs), (v) vacuolating autotransporters (VATs), and (vi) autotransporters of unknown function. The PATs, like Pet, Ssp, or EspP, could belong to different peptidase families, essentially the (i) serine peptidases S1 (IPR001314), S6 (IPR000710) or S8/S53 (IPR000209), (ii) cysteine peptidase C1 (IPR025660), or (iv) metallopeptidases M10 (IPR011049) or M28 (IPR007484). SAATs systematically feature an adhesion domain of ATs (IPR030930) like AIDA or Ag43. PHATs mainly belong to either the (i) phosphatidic phosphatase (IPR000326), (ii) tyrosine phosphatase (IPR029021), or (iii) glycoside hydrolase (IPR002772) family. LEATs, like EstA, possess GSDL lipase/esterase (IPR001087) and/or SGNH esterase (IPR013830) domains. Like VacA, the VATs systemically exhibit a vacuolating cytotoxin domain (IPR004311). The most striking observation was that no AC could be identified in any of the LEATs or VATs, whereas it was identified in the large majority of the SAATs (83%). Among the PATs and PHATs, an AC was present or absent from some functional subcategories, e.g. the M28 metalloproteases or the tyrosine phosphatases. Regarding the ATs for which no function could be inferred, homology to pectin lyase fold (IPR011050), pertactin P69 (IPR004899; IPR003991) and/or P22 tailspike protein (IPR012332) was predicted in the passengers. These regions correspond to β-helix topologies as encountered in most ATs (Jenkins et al., 1998), including the SAATs. Interestingly, all passengers belonging to the LEATs, as well as to the M28 metalloprotease or the tyrosine phosphatase ATs, exhibit α-helical folds for which no AC could be identified. Of note, the passenger of Ssp was predicted to essentially display only α-helices, like EstA, for which no AC structure could be identified either (Figure 4). Following structural modeling of the passengers, it appeared that the AC is systematically associated with passenger exhibiting β-helix folds, e.g. as observed in the passengers with a resolved structure, namely Ag43, EspP, Hap, Hbp, IcsA, IgA1, pertactin P69, and Pet, or in predicted β-helical passengers where an AC was experimentally identified, namely BrkA and AIDA-I. Despite predominantly exhibiting a single-stranded right-handed parallel β-helix in the passenger, no AC could be identified in VacA or any VATs, indicating that such as a fold is not always associated with an AC domain. Considering the oval (O), triangular (T) or L-shaped coil cross-sections, 16 different β-solenoids are currently recognised (Kajava and Steven, 2006). As expected, no O1 or O2 repeat could be identified in any of the AT passengers as these coils occur upon oligomerisation, as observed in trimeric ATs belonging to the T5cSS. Besides L1, L3, T4, T5, and T6-type β-solenoids previously reported as specific to AT passengers, some coils originally considered as specific to the T5bSS, i.e., the TPS (two-partner system), were also identified and encountered in association with an AC, namely the L2, T1, and T8 coils. Rather than being associated to some specific functions, the AC thus appears exclusively associated with β-helical passengers.

## Discussion

While the function of the AC for proper passenger secretion and folding on the cell-surface has been well demonstrated in several ATs, its commonality in the T5aSS was recently questioned. This ambiguity partly results from the difficulty in identifying the AC in uncharacterised ATs following similarity search by sequence alignment. Following a structural approach, the AC domain was here identified, first in other ATs whose passenger structure was resolved and subsequently in ATs from a recognised reference dataset (Celik et al., 2012). It must be stressed that the prediction was based on similarity of the ACs, including functionally characterised ACs, namely EspP (Velarde and Nataro, 2004), Hbp (Soprova et al., 2010), IcsA (May and Morona, 2008), and Pet (Dutta et al., 2003). It further provides the proof-of-principle that a predicted AC can indeed be functional since the AC structure was here identified in BrkA and AIDA-I, which AC domains were functionally demonstrated but not structurally resolved (Oliver et al., 2003; Berthiaume et al., 2007). It is worth mentioning that some other regions in the C-terminal half of the β-helical passengers could contribute to folding and secretion (Drobnak et al., 2015); They might function in conjunction with the AC of ATs that have them. Nevertheless and contrary to what previously believed (Drobnak et al., 2015), this investigation clearly demonstrates that the AC corresponds to a conserved structural element present in the passenger of numerous, but not all, ATs. Besides, the AC appeared to be systematically and exclusively associated to passengers exhibiting single-stranded right-handed parallel β-helix, whenever the coils belong to the L1, L2, L3, T1, T4, T5, T6, or T8-type β-solenoids. The C-terminal region of the passenger, encompassing the AC, is involved in the initiation of folding of the passenger and prevent its unfolding once formed (Junker et al., 2006; Soprova et al., 2010; Renn et al., 2012; Baclayon et al., 2016). In Hbp, the stacking of aromatic residues was found to be important for its folding and stability (Baclayon et al., 2016). Considering the current proposed model mechanism, where the AC provides the first β-helical rung to promote folding of the passenger at the cell surface after emerging from the translocator, it makes sense that the AC is prevalent and even restricted to passenger with a β-helical architecture. A priori, it is almost impossible to predict the chirality of a β-helical structure since both right- and left-handed β -helices can be expected (Kajava et al., 2001). Because it triggers the right-handed arrangement, the presence of an AC domain unambiguously indicates that the upstream region has a right-handed β-helix. While the AC is generally located at the vicinity of the translocator, it is not always the case as already reported in IgA1, where it is present at another C-terminus part of the passenger that results from post-secretional processing (Oliver et al., 2003). This observation applies to all ACs identified in cluster P, which regroup members of the IgA1 AT family. While we found additional ACs at a significant distance from the translocator (Table 1S), more thorough investigations are needed to demonstrate whether they are subjected to similar processing.

While all ACs were found exclusively associated to β-helical passengers, not all passengers with a β-helix fold seem to possess an AC. Unexpectedly, an AC domain was not identified in any of the VATs, which are still predicted to have a single-stranded right-handed parallel β-helix just like in VacA (Gangwer et al., 2007). This suggests that either an AC with a different fold is present in the VATs and could not be identified by our approach or the secretion and folding of VacA-like ATs do not require any kind of AC. Thus, although the co-existence of β-helical domain and AC has generally been assumed in ATs, this work provides for the first time evidence that this relationship is not always straightforward. Interestingly, no AC could be identified in the AT passengers predicted to have an EstA like globular fold, dominated by α-helices and loops (Van Den Berg, 2010). Unexpectedly, the Ssp passenger was predicted with a high confidence level (100.0%) to display some α-helical folds where no AC structure could be identified (Figure 4). Actually, it is in this AT that a region with an intramolecular chaperone function was for the first time reported (Ohnishi et al., 1994). Interestingly, the region corresponding to the AC domain displayed an α-fold and not a β-fold like for the AC presently identified. While the secondary structures in α-helices are predicted with a high confidence level in the S_646_–G_716_ region reported as a functional AC (Ohnishi and Horinouchi, 1996), it must be stressed the tertiary structure of this region is essentially modeled *ab initio* and is unreliable; Intensive structural modeling was undertaken but failed to provide any significant tertiary structure prediction for this AC domain in Ssp. With no crystallographic data available, the structural nature of this region remains unknown but clearly differs from that of the ACs here reported. Besides stressing the need to experimentally resolve the three-dimensional structure of the AC^Ssp^, this result pinpoints the need to experimentally determine the fold of passengers with architectures alternative to the single-stranded right-handed parallel β-helix. So far, EstA is the only AT with an α-helical passenger, which structure has been experimentally resolved (Van Den Berg, 2010). Besides the LEATs, however, such α-helical fold would occur in some PHATs and PATs (e.g. Ssp) as well. With such a gap of knowledge, we can only hypothesise on the possibility to have an alternative AC of type 2 (AC-2) with an α-fold associated to α-helical passenger as suggested by the investigation in Ssp; this would make the pair with the AC of type 1 (AC-1) presently investigated, which has a β-fold and is associated to β-helical passenger. Beyond the mechanistic aspects of the T5aSS, such information about the importance of these AC-1 and AC-2 in secretion and folding of the passenger is of great importance for biotechnological applications, e.g. aiming at efficiently expressing heterologous proteins for cell-surface display via the T5aSS (Van Ulsen et al., 2014; Nicolay et al., 2015), or for biomedical applications, e.g. aiming at blocking the AT secretion or folding to mitigate the virulence levels of pathogenic bacteria (Bondarenko et al., 2002; Wells et al., 2007).

## Author contributions

MR-L, MZ, and MD conceived and designed the experiments. MR-L, MZ, LK, XB, and AK performed the experiments and data acquisition. MR-L, MZ, LK, XB, AK, IH, FP, MP, RR, and MD analysed and interpreted the data. MR-L, LK, XB, AK, FP, MP, RR, and MD contributed to materials and analysis tools. MR-L, MZ, LK, XB, AK, IH, FP, MP, RR, and MD wrote the article, including drafting and revising critically the manuscript for important intellectual content.

### Conflict of interest statement

RR and MP are permanent employees of GSK, which provided support in the form of salaries. The other authors declare that the research was conducted in the absence of any commercial or financial relationships that could be construed as a potential conflict of interest.
